# Sublingual ranulas, is it time for a new classification? A systematic review and meta-analysis

**DOI:** 10.1017/S0022215124001464

**Published:** 2025-02

**Authors:** Matteo Lazzeroni, Massimo Del Fabbro, Michele Gaffuri, Mark McGurk, Gabriele Alessandro Novarria, Gianluca Martino Tartaglia, Lorenzo Pignataro, Pasquale Capaccio

**Affiliations:** 1 Department of Otorhinolaryngology & Head and Neck Surgery, Amsterdam UMC, University of Amsterdam, Amsterdam, The Netherlands; 2 Department of Biomedical, Surgical and Dental Sciences, University of Milan, Milan, Italy; 3 Department of Otorhinolaryngology & Head and Neck Surgery, Fondazione IRCCS Ca’ Granda Ospedale Maggiore Policlinico, Milan, Italy; 4 Department of Clinical Sciences and Community Health, University of Milan, Milan, Italy; 5 Head and Neck Academic Centre, Department of Head and Neck Surgery, University College London Hospital, London, United Kingdom; 6 Department of Otorhinolaryngology & Head and Neck Surgery, Fatebenefratelli Hospital, ASST Fatebenefratelli Sacco, Milan, Italy

**Keywords:** mouth floor, oral surgical procedures, ranula, salivary glands, sublingual gland

## Abstract

**Objectives:**

Sublingual ranulas present diagnostic and therapeutic challenges due to their heterogenous clinical presentations. This systematic review and meta-analysis aims to synthesise treatment outcomes and proposes a new classification for this condition.

**Methods:**

Following PRISMA guidelines, a thorough literature search identified studies on patients with sublingual ranulas receiving medical or surgical treatment. Proportion meta-analysis compared success rates among studies using a random-effects model.

**Results:**

Forty-two studies were included, covering 686 endoral ranulas, 429 plunging ranulas, and 16 ranulas extending into the parapharyngeal space. Sublingual sialoadenectomy with or without pseudocyst wall excision showed low heterogeneity and the highest success rates. Consequently, a new classification system is proposed categorising ranulas by intraoral (Type 1), cervical (Type 2) or parapharyngeal space (Type 3) extension.

**Conclusion:**

This study confirms the role of sublingual gland resection as standard of care and highlights the need for a revised classification to improve patient outcomes.

## Introduction

A sublingual ranula is an extravasation mucocele that arises from ruptured acini or ducts of the sublingual gland.^1,2^ Ranulas can only occur from sublingual or minor salivary glands, which are able to produce saliva against a pressure gradient. Major glands downregulate salivary production if obstructed.^3,4^ The submandibular and parotid gland generate a discontinuous secretory flow driven by nervous stimulation, whereas the sublingual gland spawns a continuous, spontaneous secretion of saliva, not strictly dependent of food intake.^1^ Consequently, when a leak develops within its drainage system, it continues to secrete saliva through the breach. This phenomenon is most effective in areas where the surrounding tissues are loose and lax, such as the floor of the mouth, and is less effective in regions like the hard palate where tissues are more rigid.

Sublingual ranulas are typically acquired, post-traumatic conditions.^5^ They can arise from incidental damage caused by mastication, dental implantation or iatrogenic manoeuvres,^6^ yet their aetiology remains often unclear,^7^ especially in case of unnoticed mechanical trauma to the gland. The so-called “congenital” sublingual ranula in newborns and infants, with 14 cases reported in current literature,^8–11^ is the consequence of mucus retention and extravasation from duct atresia, acinus dilatation, ostia stenosis or imperforated sublingual salivary gland.

Ranulas have traditionally been classified as simple or endoral, when confined to the oral floor, or plunging, when the pseudocyst extends into the neck, usually in the submandibular space, through a hiatus of mylohyoid muscle or behind the posterior border of the mylohyoid muscle.^3,12^

Simple ranulas are common during the first and second decade of life,^3^ while plunging ranulas occur frequently during the third decade of life, with a higher prevalence in specific ethnic groups. For this reason, a genetic predisposition for the development of plunging ranulas has been proposed in relation to the prevalence of mylohyoid defects and sublingual gland herniations in the cervical region.^13,14^

Cornerstone of the diagnostic algorithm for sublingual ranula is clinical examination, involving inspection and palpation.^12^ Radiologic assessment can be useful for differential diagnosis with other cervical space occupying lesions and for treatment planning, especially for recurrent ranulas. Ultrasonography can be considered a valid first choice examination, since it has shown accuracy in characterising ranulas regardless of their dimensions and can easily determine their possible extension in the surrounding spaces.^15^ Second choice examinations are computed tomography (CT) and magnetic resonance imaging (MRI), in which the presence of the “tail sign” is pathognomonic for plunging ranulas.^16^ When imaging is not conclusive, aspiration of the ranula's content and its testing for amylase to assess the likelihood of salivary origin can be pursued.^17^

Treatment strategies for sublingual ranulas have been a debated issue even in recent years.^18^ Complete resection of the sublingual gland is considered the most effective therapeutic strategy for this condition regardless of its extension to the surrounding regions due to its pathogenesis,^1,12^ yet, this is an invasive procedure, not free of serious complications, such as nerve injury, bleeding, infections and damage to Wharton's duct.^19^ Over time many conservative, minimally invasive techniques have been proposed to treat ranulas by means of marsupialisation^3,20^ or of injection of sclerotic drugs capable of inducing fibrosis to seal the mucous leak.^21^ The study by Chung *et al*.,^19^ in line with previous reviews,^1^ is to the best of our knowledge the only meta-analysis that has tried to synthetise and analytically compare the results of different therapeutic options available for sublingual ranulas.

Most patients with this condition are generally young^22^; therefore, the therapeutic goal has been focused on reducing treatment invasiveness. In recent years, several innovative approaches have been proposed for sublingual ranulas,^3,23^ especially regarding the use of sclerosing agents^21,24^ or botulinum toxin therapy.^25^ Given these developments, our objective is to provide an updated quantitative analysis of the results from these studies.

Heterogeneity, as in the treatment spectrum for ranulas, arises when there is a lack of uniformity in thought. Creating a systematic approach to the diagnostic-therapeutic process can be useful to harmonise data. The aim of this study was to make progress in this direction, towards systematisation, proposing a new anatomical classification and synthetising the results of different surgeons in treating this condition.

## Methods

The present systematic review was registered to the PROSPERO database (registration number CRD42023433994). The reporting of this study is in accordance with PRISMA statement^26^ and followed the guidelines in the Cochrane Handbook for Systematic Reviews of Interventions.^27^

### Population, Intervention, Comparison, Outcomes, Type of study design, Time of follow-up criteria

The PICOTT criteria for the present review were as follows: P: patients with sublingual ranula; I: different medical and surgical treatments for sublingual ranula: sclerotherapy, micro-marsupialisation, marsupialisation, sublingual sialoadenectomy, excision of the pseudocyst wall or simple aspiration of the ranula, transcervical approaches; C: not applicable; O: success rates in terms of recurrences, complication rates. Elaboration of a new classification for sublingual ranulas; T: observational and randomised studies with minimum five patients; T: mean follow-up time of minimum six months.

### Search strategy and data extraction

Systematic searches were conducted for English written studies published until the search date that reported rates of recurrences and complications after surgical or medical treatment for sublingual ranulas.

PubMed, Web of Science and Scopus databases were searched using as search strategy “sublingual ranula” on November 2, 2023. Abstracts and full texts were reviewed in duplicate by two different authors (M.L. and M.G.). To maximise the rate of inclusivity in the early stages of the review, at the abstract stage, all studies deemed eligible by at least one rater were included. Then, during the full-text review stage, disagreements were resolved by consensus between raters.

Inclusion criteria were: patients with sublingual ranulas undergoing medical or surgical therapy; age range of 1–100 years; follow-up time of a minimum period of six months; studies involving human subjects only; accurate reporting of post-operative complications, recurrence rate and of the anatomical extension of each sublingual ranula considered in relation to the outcomes described; and observational or randomised studies with a minimum of five patients.

For each study the following information was acquired: name and country of origin of first author, year of publication, study design (observational, randomised), number of patients included, mean age of the enrolled patients, radiological examinations used for diagnosis, localisation of the ranula (intraoral, plunging, extended to the parapharyngeal space), primary treatment, success rates (success = recurrence free patient after six months of treatment) and complication rates. In accordance with previous literature,^19^ treatments were categorised as: resection of sublingual gland (including partial or total resection of the sublingual gland by means of traditional or robotic approaches), excision of ranula alone or aspiration of ranula's content, sclerosing injections, transcervical approaches and/or submandibular sialoadenectomy, marsupialisation, micro-marsupialisation (for all types of suture-based techniques that did not remove the overlying mucosa of the ranula). The complications that were considered relevant for the present review were transient or permanent nerve injuries, formation of a haematoma or sialocele, infection, or injury to Wharton's duct.

### Risk of bias assessment

Two reviewers have independently assessed the risk of bias (ROB) through the appropriate JBI critical appraisal checklist tool. Disagreements between reviewers’ judgements were resolved by discussion until a consensus was achieved.

### Strategy for data synthesis

The main outcomes were the proportion of success and complications after intervention. Proportion meta-analysis was used to address them effectively, using a random-effects model. If at least two comparative studies comparing the same treatments were identified, pairwise meta-analysis was performed, using the random effects model in the presence of significant heterogeneity, otherwise the fixed effects model was used. The results were presented in the form of Forest plots. Heterogeneity was assessed using the Cochran Q and the I2 tests. For undertaking meta-analysis, STATA 17.0 software was used.

## Results

### Study selection and baseline characteristics

**Figure 1.**
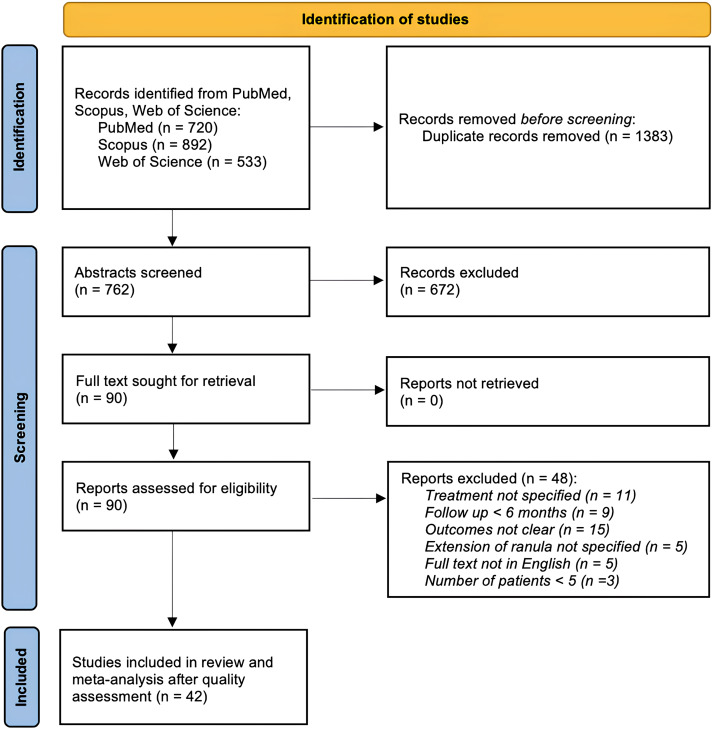
PRISMA flow diagram for the papers’ selection process.

Figure 1 reports the PRISMA flowchart of the study selection process. A total of 762 records were retrieved from PubMed, Web of Science and Scopus. After abstract screening, 90 studies were deemed eligible for full text examination. Lastly, 42^3,28–68^ studies were judged fit for the present meta-analysis according to inclusion criteria. Only one randomised control trial was found, while the others were all observation studies. The selected studies included a total of 686 endoral ranulas, 429 plunging ranulas and 16 ranulas extending into the parapharyngeal space. Detailed information about studies’ characteristics can be found in Supplementary Table 1.

### Pooled analyses of all studies and subgroup analysis

**Figure 2.**
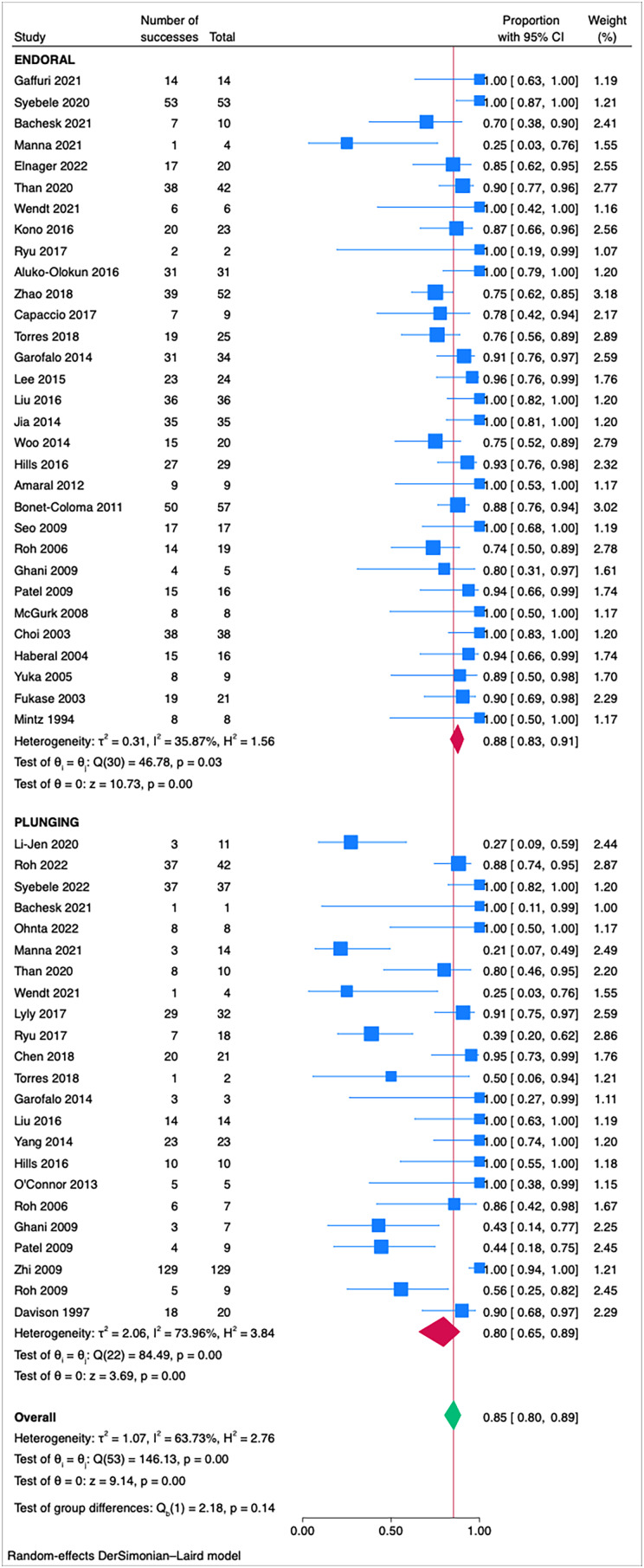
Forest plot for the overall success rates of treatment strategies for endoral - type 1 and plunging - type 2 ranulas.

As shown in Figure 2, no statistically significant differences (*p* = 0.14) were found between the success rates of treatment strategies for endoral and plunging ranulas, although effect size for plunging ranula was 0.80 (95 per cent confidence interval [CI] 0.65-0.89; I2 = 73.96 per cent), while the effect size for intraoral ranula was slightly higher at 0.88 (95 per cent CI 0.83-0.91; I2 = 35.87 per cent). Intraoral sublingual ranulas showed a tendency for better success rates and more homogeneous results compared to plunging ranulas, which instead showed more heterogeneity.

Subgroup analysis was conducted for treatment strategies that were sufficiently described in three or more separate studies to ensure an adequate level of evidence for comparative assessment.

**Figure 3.**
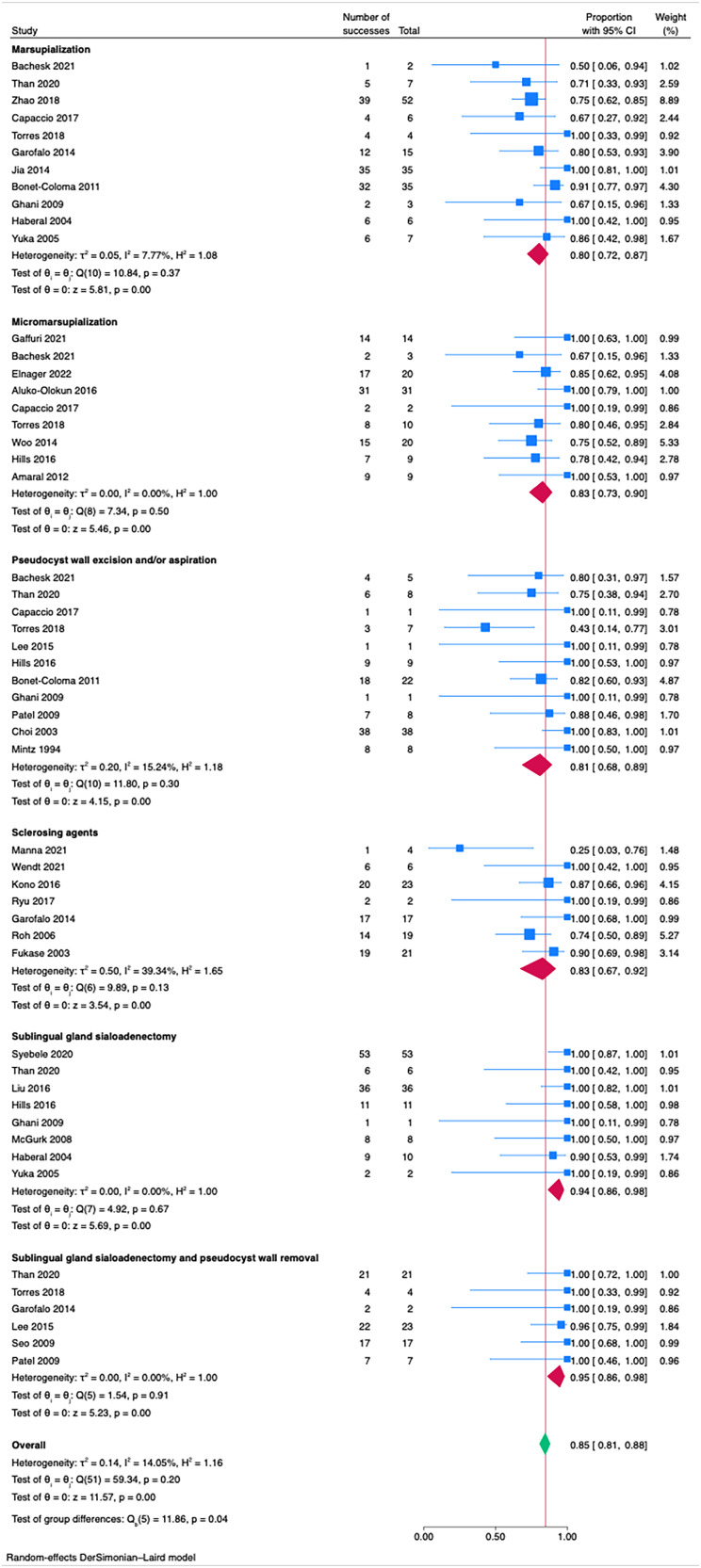
Forest plot for treatment strategies for endoral (type 1) ranulas.

Regarding endoral ranulas a global effect size of 0.85 (95 per cent CI 0.81-0.88; I2 = 14.05 per cent) across all studies was observed (Figure 3), indicating a high overall success rate. Sublingual sialoadenectomy with or without pseudocyst walls removal have shown the best success rates with an effect size of 0.95 (95 per cent CI 0.86-0.98; I2 = 0.00 per cent) and 0.94 (95 per cent CI 0.86-0.98; I2 = 0.00 per cent), respectively. The heterogeneity within the two groups was also very low, denoting highly predictable treatment outcomes. Instead, marsupialisation techniques had a wider range of success rates and an effect size of 0.80 (95 per cent CI 0.72-0.87; I2 = 7.77 per cent), indicating lower and less predictable success rates. Statistically significant differences between the groups were observed (*p* < 0.05).

**Figure 4.**
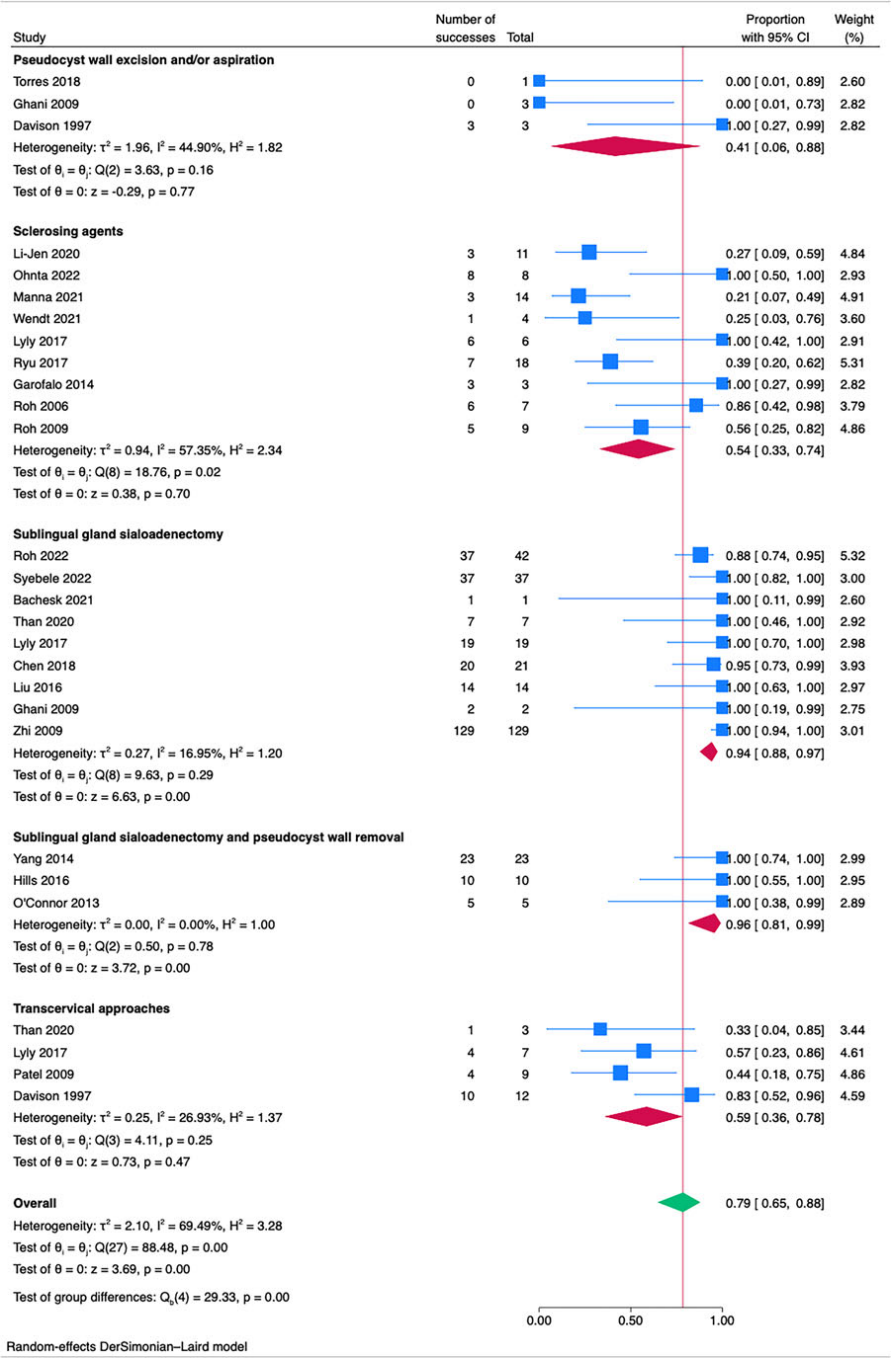
Forest plots for treatment strategies for plunging (type 2) ranulas.

Figure 4 shows results of treatments for plunging ranulas with a global effect size of 0.79 (95 per cent CI 0.65-0.88; I2 = 69.49 per cent), indicating lower overall success rates for plunging ranulas compared to simple endoral ranulas. Statistically significant differences are observed between the groups (*p* < 0.05). Heterogeneity within different treatments is variable, with sublingual sialoadenectomy with or without pseudocyst wall excision showing low heterogeneity (I^2^ = 0.00 per cent and I^2^ = 16.95 per cent, respectively), suggesting consistency and reproducibility of the results. In contrast, sclerotherapy has shown a high heterogeneity (I^2^ = 57.35 per cent) and an effect size of 0.54 (95 per cent CI 0.33-0.74), below the overall effect size.

Subgroup analysis for ranulas extending to the parapharyngeal space and for complication rates was deemed unfeasible due to the limited numbers reported in the studies included in this review, in order to avoid overinterpretation of data with insufficient statistical power.

### Quality assessment

According to the JBI critical appraisal tool (Supplementary Table 2) 17 articles were rated as low risk of bias, 15 as moderate, 5 as serious and 4 as critical. Risk of bias assessment for the only randomised controlled trial can be appreciated in Supplementary Table 3.

### Classification for sublingual ranulas

To address the heterogeneity observed in the clinical presentations of sublingual ranulas, this review proposes a novel classification system for this condition. This system aims to further specify the extension of ranulas at three main anatomical levels: intraoral (Type 1), cervical (Type 2) and parapharyngeal space (Type 3). Each type is then divided into ‘a’ and ‘b’ categories, designating further specific extensions within these anatomical regions: Type 1a - simple endoral unilateral sublingual ranula; Type 1b - simple endoral sublingual ranula with extension to the contralateral oral floor; Type 2a - sublingual plunging ranula that reaches the cervical region from a hiatus of the mylohyoid muscle; Type 2b - sublingual plunging ranula that reaches the cervical region from the posterior margin of the mylohyoid muscle; Type 3a - extended sublingual ranula involving the parapharyngeal space; Type 3b - extended sublingual ranula involving the parapharyngeal space, masticatory space and/or the infratemporal fossa.

## Discussion

Treatments for plunging ranulas showed an overall effect size of 0.80 (95 per cent CI 0.65-0.89; I2 = 73.96 per cent), while treatments for endoral ranulas showed a slightly higher effect size of 0.88 (95 per cent CI 0.83-0.91; I2 = 35.87 per cent). This indicates that, although both treatment approaches are effective, those for endoral ranulas may be marginally superior, suggesting that treatments may yield better outcomes when the ranula's extent is more limited. For the treatment of plunging ranulas, we observed high effect sizes for sublingual sialoadenectomy with or without sialoadenectomy, respectively, 0.96 (95 per cent CI 0.81-0.99; I2 = 0.00 per cent) and 0.94 (95 per cent CI 0.88-0.97; I2 = 16.95 per cent), suggesting efficacious, consistent and predictable treatment outcomes.

The use of sclerosing agents, particularly for plunging ranulas (0.64; 95 per cent CI 0.37-0.88; I2 = 79.32 per cent), was not supported by our findings as an effective treatment modality; therefore, despite the ongoing research,^24,28,33,36,38^ the use of sclerosing agents does not seem to be recommended in the treatment of this pathology. The same applies to transcervical treatments that showed a low effect size of 0.59 (95 per cent CI 0.36-0.78; I2 = 26.93) and are in line with current literature.^1,18,19^ Concerning the array of minimally invasive treatment options for endoral ranulas, marsupialisation techniques also showed less satisfactory outcomes (0.80; 95 per cent CI 0.72-0.87; I2 = 7.77 per cent), emphasising the need for careful selection of treatment based on individual patient scenarios, particularly when general anaesthesia poses a risk.

As confirmed in this meta-analysis, effective treatment of intraoral and plunging ranulas is primarily based on sublingual sialadenectomy, which yields excellent results and grants favourable outcomes.

In current literature, there are reports of extensive sublingual ranulas that not only invade the cervical region,^69^ through a hiatus of the mylohyoid muscle or its posterior margin,^15^ but also extend into the parapharyngeal space^50^ and against gravity, towards the cranial base^70^ or the infratemporal fossa.^71^ Our results suggest that success rates of treatments for sublingual ranulas are not statistically different in relation to the extension of pathology from the oral floor (Figure 2); however, the analysis revealed considerable overall heterogeneity (I2 = 63.73 per cent), with treatments for cervical ranulas showing slightly lower success rates.

The challenge in treating ranulas arises especially in the complex cases mentioned earlier, where literature is still lacking, and further contributions are needed to confidently determine the best treatment in an evidence-based medicine perspective. Considering the variability in disease presentation and treatment options, we believe that it may be time for a new, comprehensive classification of this pathology. Classification attempts are always subject to a certain imprecision, yet proposing a terminology that comprises all the possible clinical presentations of this condition could prove useful for education, sharing information and comparing results.

The limitations of this study include the potential presence of significant heterogeneity among the included studies. The relatively high I2 values of the present meta-analysis may reflect substantial variations in study protocols, sampled populations and treatment modalities. These factors could affect the results of the present work and impose caution in their interpretation. More studies on extensive ranulas, classified as Type 3 according to the present classification, are needed to assess the safety and efficacy of different treatment modalities.

## Conclusion

The present study has synthetised the different success rates of treatments for sublingual ranulas. Surgical interventions, particularly sublingual gland resection, have been confirmed as the most effective, demonstrating high success rates with low heterogeneity. The limited data precluded subgroup analysis for parapharyngeal space involvement, indicating a need for further research. The proposed new classification aims to standardise treatment approaches and facilitate clearer communication among clinicians, ultimately improving patient care. Future studies should focus on extensive ranulas to determine the safety and efficacy of different treatment modalities within an evidence-based framework.

## Supporting information

Lazzeroni et al. supplementary material 1 Lazzeroni et al. supplementary material

Lazzeroni et al. supplementary material 2 Lazzeroni et al. supplementary material

Lazzeroni et al. supplementary material 3 Lazzeroni et al. supplementary material

## Supplementary material

The supplementary material for this article can be found at https://doi.org/10.1017/S0022215124001464.
