# Supplementary figures and images for: Sublingual ranulas, is it time for a new classification? A systematic review and meta-analysis
Source: J Laryngol Otol. 2024 Oct 22;139(2):88–94. doi: 10.1017/S0022215124001464 (PMC12303724; doi:10.1017/S0022215124001464)

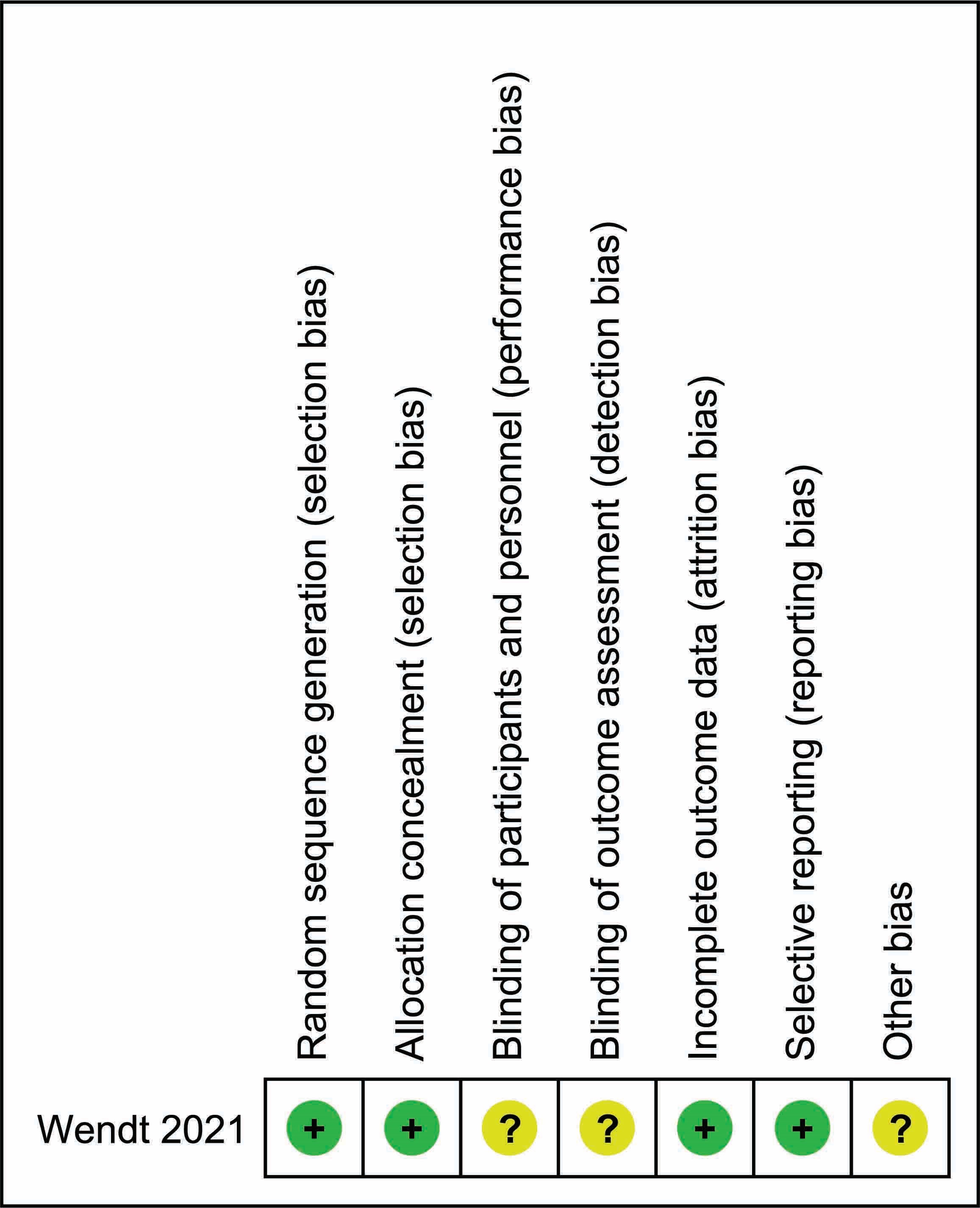

Supplement: Lazzeroni et al. supplementary material 3 — Lazzeroni et al. supplementary material [file S0022215124001464sup003.jpg]
